# Tight glucose control managed by ICU nurses induces extremely low rates of hypoglycemia

**DOI:** 10.1186/cc9815

**Published:** 2011-03-11

**Authors:** F Delodder, C Joseph, P Maravic, T Szostek, MD Schaller, M Berger, JP Revelly, P Eggimann

**Affiliations:** 1 CHUV, Lausanne, Switzerland

## Introduction

Recent studies failed to confirm survival benefit of tight glucose control (TGC). Moreover, they reported high rates (6 to 18%) of hypoglycemia (<2.5 mmol/l) associated with significant mortality. The protocols used for TGC may be difficult to apply. The reasons for blood glucose level variations are complex and TGC requires in-depth individualized knowledge of the patient condition. Frequent blood glucose measurements are mandatory for continue adaptations of insulin and glucose administration. We report the evolution of blood glucose level through various phases of TGC implementation, which become progressively completely nurse driven.

## Methods

An internal audit performed in 2002, in five of our 32 adult ICU beds, showed 26% of hyperglycemia (>10 mmol). TGC was introduced in 2003 for all patients in the ICU and supported by detailed guidelines for bedside glucose control. TGC was progressively transferred from physicians to nurses since 2007. Nurses are specifically trained to adapt infusion rates of glucose (nutrition), insulin according to medically predefined targets (4.5 to 6.0; 6.0 to 8.0; >10 mmol/l). Arterial and venous glucose levels are determined by the central laboratory or by blood gas analyzers in the ICU. Glycemia (*n *= 750,178) was extracted from our electronic clinical information system (Metavision^®^) and analyzed with STATA.

## Results

**Figure 1:**
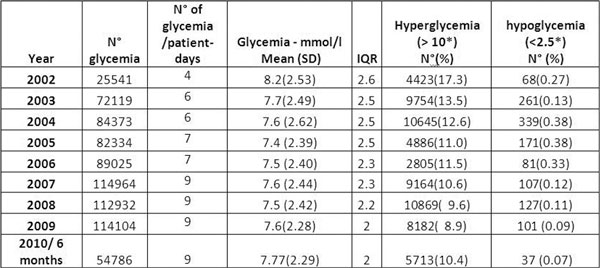


Suppression of the lowest target (4.5 to 6.0 mmol/l) in May 2009 may explain the mean increase in 2009. Improved TGC is confirmed by a continuous decrease in yearly standard deviations (IQR). The proportion of hyperglycemia decreases to less than 10% in 2008, with rates of hypoglycemia (<2.5 mmol/l) 50-fold to 100-fold lower than those reported in the literature. See Figure 1.

## Conclusions

Implementation and progressive transfer of tight glucose control to ICU nurses in a large mixed adult ICU significantly decreased the proportion of hyperglycemia to less than 10%, and maintained extremely low rates of hypoglycemia (<0.1%).

